# Lateralization Pattern of the Weber Tuning Fork Test in Longstanding Unilateral Profound Hearing Loss: Implications for Cochlear Implantation

**DOI:** 10.3390/audiolres12040036

**Published:** 2022-06-21

**Authors:** Mohamed Bassiouni, Sophia Marie Häußler, Stefan Gräbel, Agnieszka J. Szczepek, Heidi Olze

**Affiliations:** 1 Corporate Member of Freie Universität Berlin, Berlin Institute of Health, Charitéplatz 1, Humboldt-Universität zu Berlin, 10117 Berlin, Germany; stefan.graebel@charite.de (S.G.); agnes.szczepek@charite.de (A.J.S.); heidi.olze@charite.de (H.O.); 2 Department of Otorhinolaryngology, University Medical Center Hamburg-Eppendorf, Martinistraße 52, 20246 Hamburg, Germany; s.haeussler@uke.de

**Keywords:** hearing loss, tuning fork, audiometry, single-sided deafness, tinnitus

## Abstract

The Weber tuning fork test is a standard otologic examination tool in patients with unilateral hearing loss. Sound should typically lateralize to the contralateral side in unilateral sensorineural hearing loss. The observation that the Weber test does not lateralize in some patients with longstanding unilateral deafness has been previously described but remains poorly understood. In the present study, we conducted a retrospective analysis of the medical records of patients with unilateral profound hearing loss (single-sided deafness or asymmetric hearing loss) for at least ten years. In this patient cohort, childhood-onset unilateral profound hearing loss was significantly associated with the lack of lateralization of the Weber tuning fork test (Fisher’s exact test, *p* < 0.05) and the absence of tinnitus in the affected ear (Fisher’s exact test, *p* < 0.001). The findings may imply a central adaptation process due to chronic unilateral auditory deprivation starting before the critical period of auditory maturation. This notion may partially explain the poor outcome of adult cochlear implantation in longstanding single-sided deafness. The findings may suggest a role for the Weber test as a simple, quick, and economical tool for screening poor cochlear implant candidates, thus potentially supporting the decision-making and counseling of patients with longstanding single-sided deafness.

## 1. Introduction

Tuning fork tests have remained a mainstay of otologic examination for over a century. The Weber tuning fork test has been mainly used in patients with unilateral hearing loss to distinguish between sensorineural and conductive hearing loss [1,2,3,4]. In patients with conductive hearing loss, the sound should typically lateralize to the affected side, whereas in sensorineural hearing loss, it lateralizes to the contralateral side. The mechanism of sound lateralization of the Weber test has intrigued hearing health professionals for many decades [1,2,3,4]. Clinical and animal experiments have shown that bone conduction stimulates the cochlea mainly through two routes: (1) through the vibration of the middle ear ossicles and (2) vibrations of the skull itself (mainly of the cerebrospinal fluid) [4]. In the case of unilateral sensorineural hearing loss, the intercochlear intensity and phase differences lead to vibrations being perceived louder in the contralateral unaffected ear, producing sound lateralization. The observation that some patients with longstanding unilateral deafness fail to lateralize on the Weber test has been previously mentioned in the literature but remains poorly understood [2,5,6]. To date, there is no explanation as to why some patients with longstanding single-sided deafness lateralize and others do not. This article reports on thirteen cases of patients with longstanding unilateral profound hearing loss of various etiologies. These patients had different lateralization patterns on the Weber tuning fork test, seemingly related to the age of onset of deafness.

## 2. Materials and Methods

The study was approved by the ethics committee of Charité Medical University (approval number EA1/015/21). The analysis involved the retrospective review of the hospital records and audiograms of adult patients with profound unilateral hearing loss (UHL) of at least ten years’ duration who presented to our outpatient department and/or auditory implant clinic between 2018 and 2021. Pure tone audiograms (PTA) determined the ipsilateral and contralateral hearing status. Profound hearing loss was defined by a pure tone threshold average of 90 dB or higher, as described previously [7,8]. The pure tone threshold average was considered the average threshold at four frequencies: 0.5, 1, 2, and 4 kHz. The Weber tuning fork test was performed using a standard 512 Hz tuning fork for all patients. To meet the inclusion criteria, the patients had to have an audiometric interaural asymmetry of at least 50 dB at the frequency of the tuning fork tone (512 kHz). According to the status of the contralateral ear, the patients were divided into single-sided deafness (SSD) or asymmetric hearing loss (AHL) groups. SSD was defined by contralateral a PTA four-frequency average threshold below or equal to 30 dB, while AHL was defined by a PTA four-frequency average threshold above 30dB [9,10]. Statistical analysis was performed using JMP 15 Software (Statistical Analysis Systems “SAS” Institute, Cary, NC, USA).

## 3. Results

### 3.1. Patient Cohort

Thirteen adult patients met the inclusion criteria. Their age ranged from 26 to 78 years (median 54 ± 16.3 years); seven patients were male, and six were female. All patients were diagnosed with profound unilateral hearing loss (UHL) of at least ten years’ duration. The patients’ epidemiological, clinical, and medical history details are summarized in Table 1. Of the 13 patients, 8 had childhood-onset UHL and 5 had adult-onset UHL. The duration of UHL ranged from 10 to 73 years. According to the status of the contralateral ear, ten patients had single-sided deafness (SSD), while three patients had asymmetric hearing loss (AHL). Eight of thirteen patients reported having tinnitus in the poorer hearing ear.

### 3.2. Weber Test Lateralization Pattern and the Age of Onset

All but one patient with childhood-onset UHL (seven of eight patients) reported hearing the tuning fork tone during the Weber test but without lateralization to one side. In contrast, all but one patient with adult-onset UHL (four of five patients) reported lateralization to the contralateral side, and no patients lateralized to the ipsilateral side in this cohort. A contingency analysis showed a significant correlation between the age of onset (adult vs. childhood) and the pattern of lateralization of the Weber test (Fisher’s exact test, *p* = 0.0319). The patients’ age, gender, and duration of UHL did not significantly correlate with the Weber test result in this cohort.

Concerning tinnitus, 5 of 13 patients (38%) reported having tinnitus in the worse hearing ear (ipsilesional tinnitus). No patients with childhood-onset UHL reported having tinnitus, while all patients with adult-onset UHL reported having ipsilesional tinnitus. On a statistical contingency analysis, the absence of tinnitus correlated significantly with the lack of lateralization during the Weber test (Fisher’s exact test, *p* = 0.0008). Other factors such as the ipsilateral residual hearing or the contralateral hearing status were not significantly associated with the Weber test lateralization pattern in this patient cohort.

### 3.3. Case Presentations (Selected Cases)

**Figure 1:**
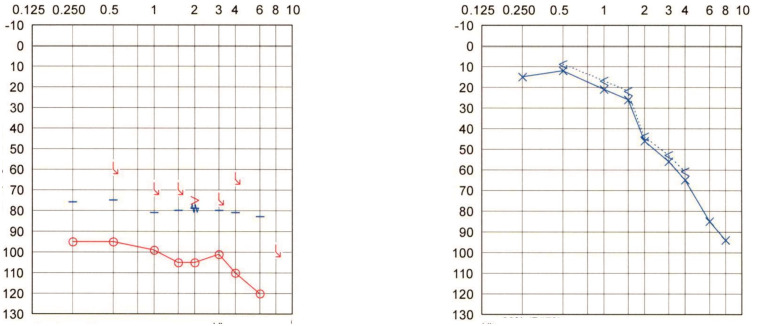
Pure tone audiogram of Patient #1 with right-sided profound hearing loss caused by scarlet fever 72 years ago.

**Patient #1:** 78-year-old woman who suffered from scarlet fever during childhood (around the age of six), resulting in a profound hearing loss on the right side. The patient reported having heard no sounds in the right ear since childhood, even in very noisy environments. The patient also denied having tinnitus. The pure tone audiogram (PTA) detected some measurable hearing in the right ear at very high intensities. The contralateral left ear showed sloping sensorineural hearing loss. The Weber test, performed with a 512 Hz tuning fork, consistently failed to indicate lateralization, as the patient has reported hearing the tone in the middle of the head. The PTA is shown in Figure 1.

**Figure 2:**
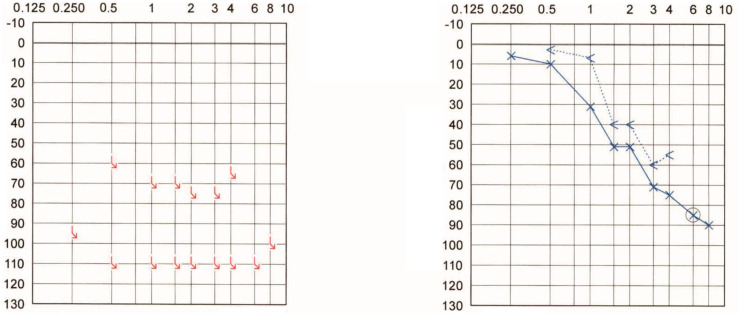
Pure tone audiogram of Patient #3 approximately 45 years after right-sided modified radical mastoidectomy with partial labyrinthectomy for extensive middle ear cholesteatoma.

**Patient #3:** 76-year-old woman who, at the age of 31, underwent a modified radical mastoidectomy for an invasive middle ear cholesteatoma on the right side. As a result of the cholesteatoma and the surgery, the patient had right-sided profound deafness and facial palsy. No recurrence of her cholesteatoma occurred in the following decades. The patient presented to the outpatient department with mastoid cavity problems. The PTA (Figure 2) indicated a sloping hearing loss in the contralateral (left) side with a small air-bone gap after a left-sided tympanoplasty decades ago. The Weber tuning fork test consistently lateralized to the contralateral (left) side, despite the very long UHL duration (45 years).

**Figure 3:**
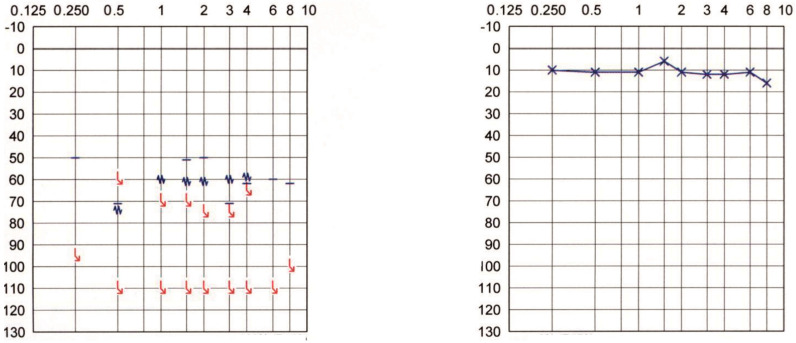
Pure tone audiogram of Patient #4 approximately 17 years after translabyrinthine surgery for right-sided vestibular schwannoma.

**Patient #4:** 54-year-old man who underwent a translabyrinthine surgery for right-sided vestibular schwannoma 17 years ago. Immediately after the surgery, the patient reported a right-sided profound hearing loss (PTA is shown in Figure 3) and facial palsy, both of which have persisted to the present day. The contralateral side retained normal hearing. The magnetic resonance follow-up scans determined no evidence of tumor recurrence, and the Weber test lateralized to the contralateral (left) side.

**Figure 4:**
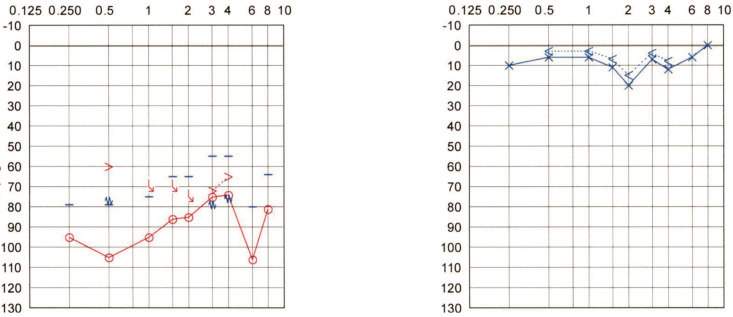
Pure tone audiogram of Patient #7 approximately ten years after right-sided sudden sensorineural hearing loss.

**Patient #7:** 56-year-old woman who suffered from an acute episode of right-sided sudden sensorineural hearing loss (SSNHL) at 46 years. The contralateral side showed normal hearing. Ten years later, the Weber tuning fork test does not lateralize, but instead, it is heard in the middle of the head. The hearing loss and tinnitus did not recover. The patient elected not to undergo cochlear implantation. The PTA is shown in Figure 4.

**Figure 5:**
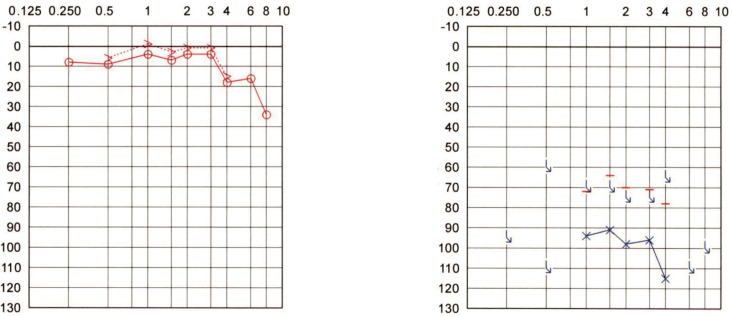
Pure tone audiogram of Patient #9 with left-sided profound hearing loss since birth.

**Patient #9:** 54-year-old man with profound left-sided sensorineural hearing loss of unknown etiology since birth. The PTA showed contralateral normal hearing (Figure 5). The Weber tuning fork test consistently showed a failure of lateralization.

**Figure 6:**
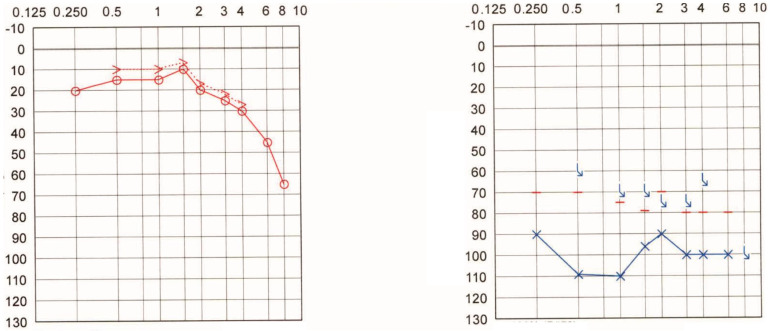
Pure tone audiogram of Patient #10 with left-sided Menière’s disease.

**Patient #10** is a 52-year-old man with left-sided Menière’s disease for over twenty years. The disease progression had resulted in recurrent vertigo attacks, left-sided sensorineural hearing loss, and tinnitus. After multiple intratympanic gentamicin injections, complete control of the vertigo attacks was reached. However, the intratympanic gentamicin treatment resulted in a left-sided profound UHL for over 18 years. The Weber test lateralized to the contralateral (right) side. The patient was referred to the cochlear implantation unit for auditory rehabilitation. The PTA is shown in Figure 6.

## 4. Discussion

In the present study, we analyzed the results of the Weber test in a small cohort of patients with single-sided deafness (SSD) and asymmetric hearing loss (AHL). The common features shared among all patients in the present study are the unilaterality and long duration of the profound hearing loss, ranging from ten to over seventy years. Despite the anticipated lateralization of the Weber test in the entire cohort, eight of thirteen patients reported the sound heard equally on both sides (no lateralization).

We hypothesize that the longstanding auditory deprivation associated with profound unilateral hearing loss (UHL) could lead to a central adaptation process, contributing to the loss of lateralization of the Weber tuning fork test. This observation appears to apply to both SSD and AHL. It is tempting to speculate that the loss of Weber test lateralization could be attributed to central habituation. Observations from daily otologic practice support such a notion. For instance, patients who underwent successful stapedotomies commonly describe environmental sounds as uncomfortably loud for a short period immediately after the surgery [1]. This observation can be explained by the central adaptation to a low-sound-intensity input from that ear over a long time. Since this discomfort is generally transient, this central adaptation appears to be reversible in those cases of chronic conductive hearing loss [1]. After an acute unilateral vestibular loss, an equivalent central habituation process is also well established [11,12,13]. It would be interesting to determine whether such an adaptation process in the central auditory system influences the outcome of cochlear implantation in patients with SSD.

The effects of unilateral auditory deprivation on the auditory cortex have been previously studied [14,15]. Children with SSD display neural plastic changes in the auditory cortex, particularly cortical reorganization and interaural preference, which may be reversible after early cochlear implantation [15,16,17,18]. Interestingly, auditory cortex maturation continues well into adolescence [19], which may explain the absence of tinnitus and lack of Weber test lateralization observed in patients with postlingual childhood-onset SSD in the present study. These cortical changes may explain the poorer outcome of cochlear implantation in prelingual SSD compared to postlingual SSD [15]. However, animal models of SSD have also shown neuroplastic changes in the subcortical auditory centers [20], making the model even more complex. This complexity renders it challenging to employ specific electrophysiologic or radiological markers as predictors for the outcome of cochlear implantation in patients with longstanding SSD. One potential approach would be to use functional magnetic resonance imaging (fMRI) to evaluate the central reorganization occurring after SSD [21,22]. Based on the current study’s findings, we suggest adding the Weber tuning fork test to the standard test battery to evaluate cochlear implant candidacy in SSD patients.

SSD patients should be counseled about the alternatives to cochlear implantation, including contralateral routing of signals (CROS) or bone conduction devices. While those devices may abolish the head shadow effect, they do not restore binaural hearing [23,24]. In the well-selected motivated SSD patient, cochlear implantation may thus be an attractive treatment option that allows binaural hearing with improved sound localization, tinnitus relief, speech discrimination in noise, and quality of life [25,26]. In a recent randomized controlled trial, cochlear implantation outperformed CROS and bone conduction devices in SSD [26]. However, the auditory outcomes of cochlear implantation in congenital and longstanding SSD still represent a challenge, which was the primary motivation behind the present study, aiming to provide clinical predictors of cochlear implant performance in longstanding SSD.

The lack of reliable electrophysiologic or radiological outcome predictors for cochlear implantation in SSD patients may reflect the incomplete understanding of the neurobiological changes associated with SSD. Indeed, the outcome of cochlear implantation in SSD is variable and depends on several factors, most notably the duration of deafness [27,28,29,30,31]. A long duration of SSD has been associated with poor cochlear implant performance and deficient postoperative speech discrimination [27,28,29,30,31]. As a result, many clinicians do not recommend cochlear implantation in longstanding SSD. However, the outcomes of auditory rehabilitation with cochlear implants vary among SSD patients, precluding a consensus or guideline, since some patients still achieve some degree of benefit even after long durations of deafness [32]. A systematic review of the literature identified a statistically significant negative association between the SSD duration and postoperative speech discrimination [27]. However, the effect size was not clear, thus not allowing for a recommendation on the longest accepted period of SSD for cochlear implant candidacy [27], especially considering the myriad of other confounding factors influencing the outcome. In our own published data on implanted adults with SSD, there was a statistically significant correlation between a longer duration of SSD and poorer postoperative implant performance, with congenital SSD patients having zero speech discrimination in the implanted ear one year after implantation [28,33].

In clinical and experimental studies, the association between tinnitus and SSD has been well established [14]. Chronic subjective tinnitus may be regarded as a central response to peripheral auditory deafferentiation [15,34]. Translational audiology experiments with animal models of SSD have demonstrated the lack of tinnitus in congenital SSD [34,35]. This finding has been confirmed by human clinical studies of congenital SSD patients, suggesting that auditory experience is essential for the development of tinnitus [36]. Furthermore, the duration of auditory experience must be sufficiently long for the development of tinnitus [37]. Indeed, Lee and coworkers reported the absence of subjective ipsilesional tinnitus in adult patients with SSD with onset before the age of 20 years [37]. These findings suggest that the lack of tinnitus in SSD may be associated with irreversible neural plastic changes if they persist after the critical point of auditory development in adolescence. As such, the absence of tinnitus in adult patients with childhood-onset SSD may indicate irreversible central changes. Future studies should investigate the potential usefulness of central auditory processing evaluations in detecting those changes. In adult-onset SSD, the role of tinnitus as a predictive factor is less clear. Previous studies have shown that the severity of tinnitus associated with sudden sensorineural hearing loss (SSNHL) decreases with time [38]. Some authors hypothesized that SSD patients with tinnitus might have better auditory outcomes after cochlear implantation than those without tinnitus [39]. In the present study, ipsilesional tinnitus was reported exclusively by patients with adult-onset UHL, which is consistent with the findings of Lee et al. [37]. In the patients with childhood-onset UHL, the absence of tinnitus correlated significantly with the lack of Weber test lateralization. Since our data are only correlative and retrospective, the findings should still be confirmed in further prospective studies. The small sample size and the heterogeneous patient cohort represent the main limitations of our research.

## 5. Conclusions

In patients with longstanding SSD referred for cochlear implantation, the poor postimplantation auditory performance can be partially explained by peripheral factors (such as spiral ganglion neurite retraction or neuronal loss). However, a central habituation component is also likely involved, as shown by electrophysiological and brain mapping studies [14,17,40]. It is tempting to hypothesize that the lack of Weber test lateralization could predict this central habituation process, possibly forecasting the auditory performance of implanted SSD patients. Based on this hypothesis, we proposed a novel role for the Weber test as a simple adjunct screening tool before cochlear implantation of SSD patients. If confirmed, this phenomenon may have implications for otologic practice, potentially supporting the decision-making and counseling of patients with longstanding SSD, who seek auditory rehabilitation. Further, more extensive studies are needed to elucidate the relation between the Weber tuning fork test result and the cochlear implant performance in patients with SSD. In addition to clinical research, the reverse translational approach (“bed-to bench”) is recommended for further studies to determine the precise cell biology- and neurobiology-based mechanisms of the changes seen in clinical practice.

## Figures and Tables

**Table 1:** Patient characteristics of the study cohort. AHL: asymmetric hearing loss; SSD: single-sided deafness; UHL: unilateral profound hearing loss. SSNHL: sudden sensorineural hearing loss. F: female; M: male.

	Age (Years)	Gender	Weber Test	Residual Hearing	SSD vs. AHL	Etiology	Age of Onset (Years)	Onset Classification	Duration of UHL (Years)	Tinnitus
#1	78	F	No lateralization	Yes	AHL	Infectious (scarlet fever)	6	Childhood onset	72	No
#2	34	M	Lateralized	No	SSD	Infectious (meningitis)	4	Childhood onset	30	No
#3	75	F	Lateralized	No	AHL	Surgery	22	Adult onset	53	Yes
#4	54	M	Lateralized	No	SSD	Surgery	37	Adult onset	17	Yes
#5	54	M	No lateralization	Yes	SSD	Trauma	7	Childhood onset	47	No
#6	54	M	No lateralization	No	SSD	Infectious (labyrinthitis)	4	Childhood onset	50	No
#7	56	F	No lateralization	Yes	SSD	SSNHL	46	Adult onset	10	Yes
#8	36	F	No lateralization	No	SSD	Congenital	0	Childhood onset	36	No
#9	54	M	No lateralization	No	SSD	Congenital	0	Childhood onset	54	No
#10	51	M	Lateralized	Yes	SSD	Menière’s disease	33	Adult onset	18	Yes
#11	44	F	Lateralized	Yes	SSD	SSNHL	22	Adult onset	22	Yes
#12	26	F	No lateralization	No	SSD	Infectious (mumps)	3	Childhood onset	23	No
#13	78	F	No lateralization	Yes	AHL	Infectious	5	Childhood onset	73	No

## Data Availability

Not applicable.

## References

[1] Huizing E.H. (1970). Lateralization of bone conduction into the better ear in conductive deafness. Paradoxical Weber test in unilaterally operated otosclerosis. Acta Otolaryngol..

[2] Guindi G.M. (1981). Lateralization of the Weber response after stapedectomy. Br. J. Audiol..

[3] Blakley B.W., Siddique S. (1999). A qualitative explanation of the Weber test. Otolaryngol. Head Neck Surg..

[4] Sichel J.Y., Freeman S., Sohmer H. (2002). Lateralization during the Weber test: Animal experiments. Laryngoscope.

[5] Scott-Brown W.G., Ballantyne J., Groves J. (1979). Scott-Brown’s Diseases of the Ear, Nose and Throat: The Ear.

[6] Ghosh P. (1995). Weber-QUO vedis?. Indian J. Otolaryngol. Head Neck Surg..

[7] Goodman A. (1965). Reference zero levels for pure-tone audiometers. J. Speech Lang. Hear. Res..

[8] Clark J.G. (1981). Uses and abuses of hearing loss classification. Asha.

[9] Vincent C., Arndt S., Firszt J.B., Fraysse B., Kitterick P.T., Papsin B.C., Snik A., Van de Heyning P., Deguine O., Marx M. (2015). Identification and evaluation of cochlear implant candidates with asymmetrical hearing loss. Audiol. Neurootol..

[10] Van de Heyning P., Távora-Vieira D., Mertens G., Van Rompaey V., Rajan G.P., Müller J., Hempel J.M., Leander D., Polterauer D., Marx M. (2016). Towards a Unified Testing Framework for Single-Sided Deafness Studies: A Consensus Paper. Audiol. Neurootol..

[11] Curthoys I.S., Halmagyi G.M. (1995). Vestibular compensation: A review of the oculomotor, neural, and clinical consequences of unilateral vestibular loss. J. Vestib. Res. Equilib. Orientat..

[12] Strupp M., Arbusow V., Maag K.P., Gall C., Brandt T. (1998). Vestibular exercises improve central vestibulospinal compensation after vestibular neuritis. Neurology.

[13] Dutia M.B. (2010). Mechanisms of vestibular compensation: Recent advances. Curr. Opin. Otolaryngol. Head Neck Surg..

[14] Gordon K., Kral A. (2019). Animal and human studies on developmental monaural hearing loss. Hear. Res..

[15] Liu J., Zhou M., He X., Wang N. (2020). Single-sided deafness and unilateral auditory deprivation in children: Current challenge of improving sound localization ability. J. Int. Med. Res..

[16] Sharma A., Glick H., Campbell J., Torres J., Dorman M., Zeitler D.M. (2016). Cortical plasticity and reorganization in pediatric single-sided deafness pre- and postcochlear implantation: A case study. Otol. Neurotol..

[17] Polonenko M.J., Gordon K.A., Cushing S.L., Papsin B.C. (2017). Cortical organization restored by cochlear implantation in young children with single sided deafness. Sci. Rep..

[18] Lee H.J., Smieja D., Polonenko M.J., Cushing S.L., Papsin B.C., Gordon K.A. (2020). Consistent and chronic cochlear implant use partially reverses cortical effects of single sided deafness in children. Sci. Rep..

[19] Yamazaki H., Easwar V., Polonenko M.J., Jiwani S., Wong D.D.E., Papsin B.C., Gordon K.A. (2018). Cortical hemispheric asymmetries are present at young ages and further develop into adolescence. Hum. Brain Mapp..

[20] Kim S.Y., Heo H., Kim D.H., Kim H.J., Oh S.H. (2018). Neural plastic changes in the subcortical auditory neural pathway after single-sided deafness in adult mice: A MEMRI study. Biomed. Res. Int..

[21] Scheffler K., Bilecen D., Schmid N., Tschopp K., Seelig J. (1998). Auditory cortical responses in hearing subjects and unilateral deaf patients as detected by functional magnetic resonance imaging. Cereb. Cortex.

[22] Bilecen D., Seifritz E., Radü E.W., Schmid N., Wetzel S., Probst R., Scheffler K. (2000). Cortical reorganization after acute unilateral hearing loss traced by fMRI. Neurology.

[23] Peters J.P., Smit A.L., Stegeman I., Grolman W. (2015). Review: Bone conduction devices and contralateral routing of sound systems in single-sided deafness. Laryngoscope.

[24] Kitterick P.T., Smith S.N., Lucas L. (2016). Hearing Instruments for Unilateral Severe-to-Profound Sensorineural Hearing Loss in Adults: A Systematic Review and Meta-Analysis. Ear Hear.

[25] Cabral Junior F., Pinna M.H., Alves R.D., Malerbi A.F., Bento R.F. (2016). Cochlear Implantation and Single-sided Deafness: A Systematic Review of the Literature. Int. Arch. Otorhinolaryngol..

[26] Peters J.P.M., van Heteren J.A.A., Wendrich A.W., van Zanten G.A., Grolman W., Stokroos R.J., Smit A.L. (2021). Short-term outcomes of cochlear implantation for single-sided deafness compared to bone conduction devices and contralateral routing of sound hearing aids-Results of a Randomised controlled trial (CINGLE-trial). PLoS ONE.

[27] Cohen S.M., Svirsky M.A. (2019). Duration of unilateral auditory deprivation is associated with reduced speech perception after cochlear implantation: A single-sided deafness study. Cochlear Implant. Int..

[28] Haussler S.M., Kopke V., Knopke S., Grabel S., Olze H. (2020). Multifactorial positive influence of cochlear implantation on patients with single-sided deafness. Laryngoscope.

[29] Kurz A., Grubenbecher M., Rak K., Hagen R., Kuhn H. (2019). The impact of etiology and duration of deafness on speech perception outcomes in SSD patients. Eur. Arch. Oto-Rhino-Laryngol..

[30] van Zon A., Peters J.P., Stegeman I., Smit A.L., Grolman W. (2015). Cochlear implantation for patients with single-sided deafness or asymmetrical hearing loss: A systematic review of the evidence. Otol. Neurotol..

[31] Blasco M.A., Redleaf M.I. (2014). Cochlear implantation in unilateral sudden deafness improves tinnitus and speech comprehension: Meta-analysis and systematic review. Otol. Neurotol..

[32] Nassiri A.M., Wallerius K.P., Saoji A.A., Neff B.A., Driscoll C.L.W., Carlson M.L. (2022). Impact of duration of deafness on speech perception in single-sided deafness cochlear implantation in adults. Otol. Neurotol..

[33] Haussler S.M., Knopke S., Dudka S., Grabel S., Ketterer M.C., Battmer R.D., Ernst A., Olze H. (2020). Improvement in tinnitus distress, health-related quality of life and psychological comorbidities by cochlear implantation in single-sided deaf patients. HNO.

[34] Knipper M., van Dijk P., Schulze H., Mazurek B., Krauss P., Scheper V., Warnecke A., Schlee W., Schwabe K., Singer W. (2020). The Neural Bases of Tinnitus: Lessons from Deafness and Cochlear Implants. J. Neurosci..

[35] Eggermont J.J., Kral A. (2016). Somatic memory and gain increase as preconditions for tinnitus: Insights from congenital deafness. Hear. Res..

[36] Lee S.Y., Nam D.W., Koo J.W., De Ridder D., Vanneste S., Song J.J. (2017). No auditory experience, no tinnitus: Lessons from subjects with congenital- and acquired single-sided deafness. Hear. Res..

[37] Lee J.M., Kim Y., Ji J.Y., Koo J.W., Song J.J. (2021). Auditory experience, for a certain duration, is a prerequisite for tinnitus: Lessons from subjects with unilateral tinnitus in the better-hearing ear. Prog. Brain Res..

[38] Muhlmeier G., Baguley D., Cox T., Suckfull M., Meyer T. (2016). Characteristics and spontaneous recovery of tinnitus related to idiopathic sudden sensorineural hearing loss. Otol. Neurotol..

[39] Liu Y.W., Cheng X., Chen B., Peng K., Ishiyama A., Fu Q.J. (2018). Effect of tinnitus and duration of deafness on sound localization and speech recognition in noise in patients with single-sided deafness. Trends Hear..

[40] Kral A., Hubka P., Heid S., Tillein J. (2013). Single-sided deafness leads to unilateral aural preference within an early sensitive period. Brain.

